# Supplementary material for: Exploratory analysis of eating- and physical activity-related outcomes from a randomized controlled trial for weight loss maintenance with exercise and liraglutide single or combination treatment
Source: Nat Commun. 2022 Aug 15;13:4770. doi: 10.1038/s41467-022-32307-y (PMC9378667; doi:10.1038/s41467-022-32307-y)
Supplement: Supplementary file 1 — Supplementary Information [file 41467_2022_32307_MOESM1_ESM.pdf]

Supplementary Appendix

Exploratory analysis of eating- and physical activity-related outcomes from a randomized controlled trial for weight loss maintenance with exercise and liraglutide single or combination treatment

Contents

Figure S1. CONSORT flow diagram 2

Figure S2. Standardized food pictures for assessment of food preferences (Leeds Food Preference Questionnaire). 3

Figure S3. Changes in postprandial appetite suppression and eating behavior during the study in the intention-to-treat population. 4

Figure S4. Changes in sedentary time during the study in the intention-to-treat population. 5

Table S1. Differences in change in outcomes between the three active weight maintenance groups versus placebo from randomization to week 52........ 6

Table S2. Differences in change in outcomes between the three active weight maintenance groups versus placebo from randomization to week 52 in the intention to treat population. 7

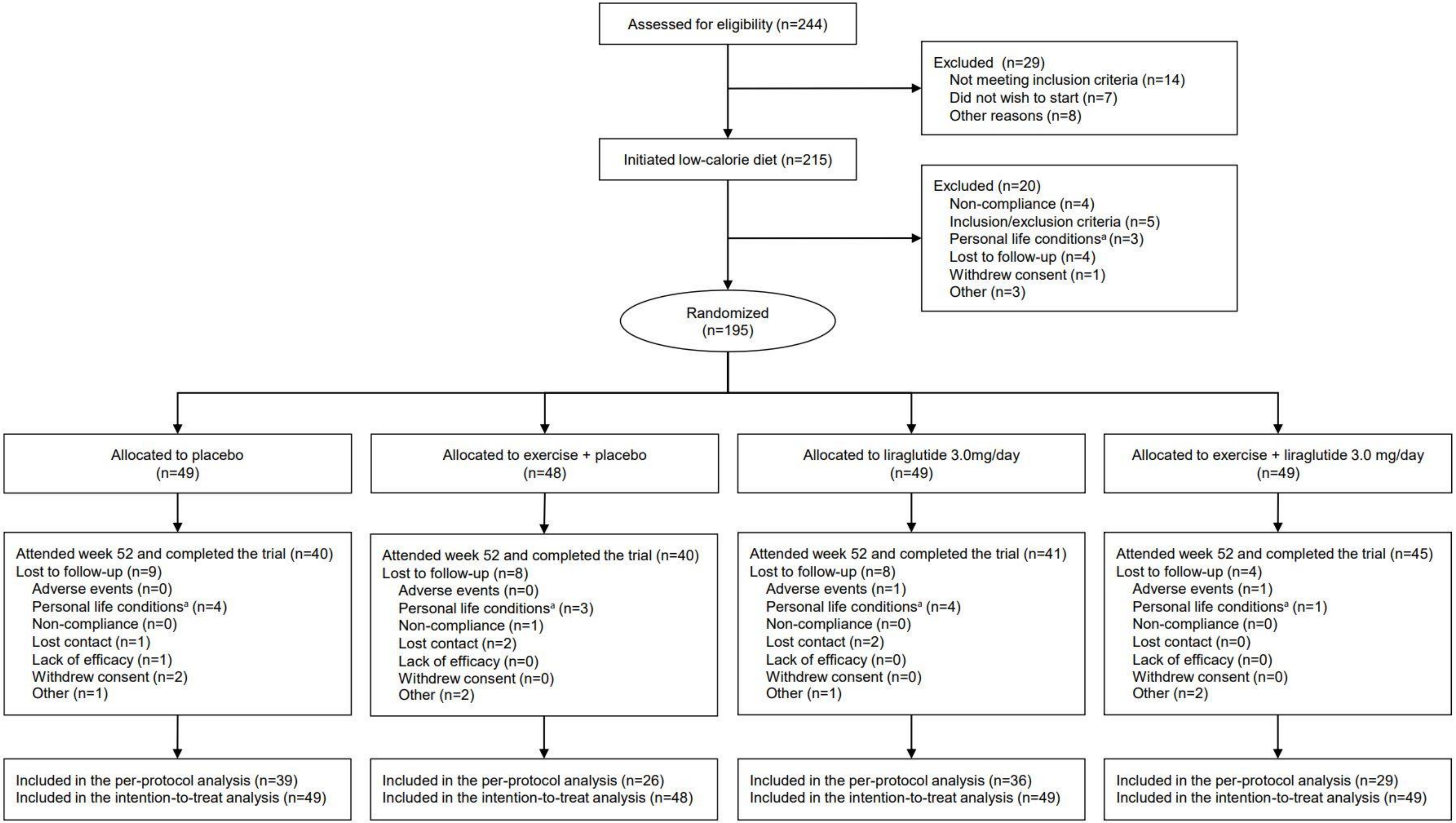

Figure S1. CONSORT flow diagram. [a] personal life conditions could be job-related, moving, disease, or death in the family.

	High fat	Low fat
**Sweet**	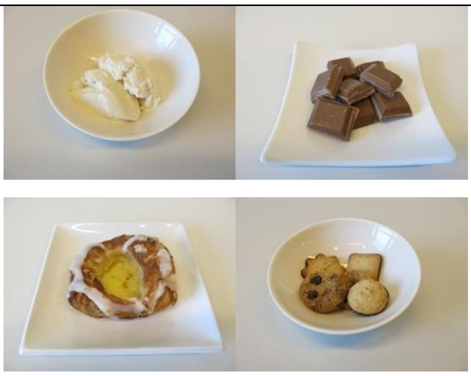	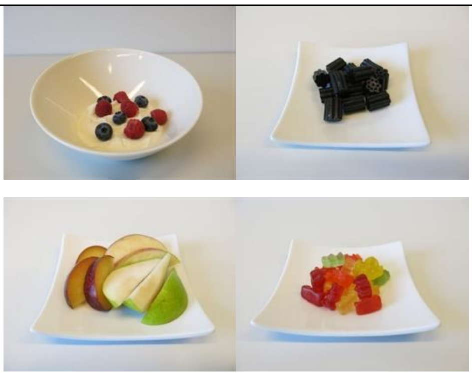
**Savory**	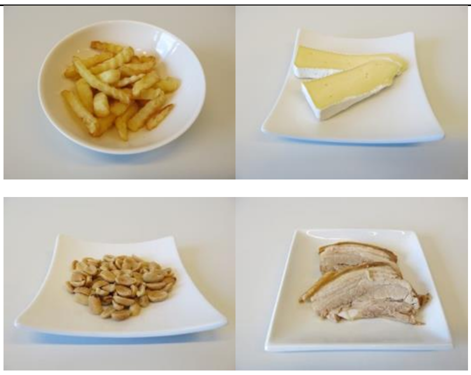	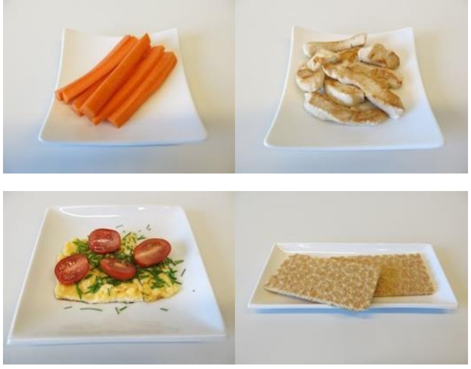

Figure S2. Standardized food pictures for assessment of food preferences (Leeds Food Preference Questionnaire). Food preferences and food reward responses, i.e., liking and implicit wanting of food items, were measured in fasted state by a computerized task where standardized pictures of typical Danish food items were shown in the categories of High Fat Savory, Low Fat Savory, High Fat Sweet and Low Fat Sweet (pork rib roast, French fries, nuggets, salty crackers, cheese, smoked fillet (cold cuts), omelet, carrots, crisp bread, turkey strips, Danish pastries, vanilla ice cream, milk chocolate, pound cake, cookies, cut fruits, skyr (yoghurt) with berries, sweet licorice, cocoa meringues and wine gum).

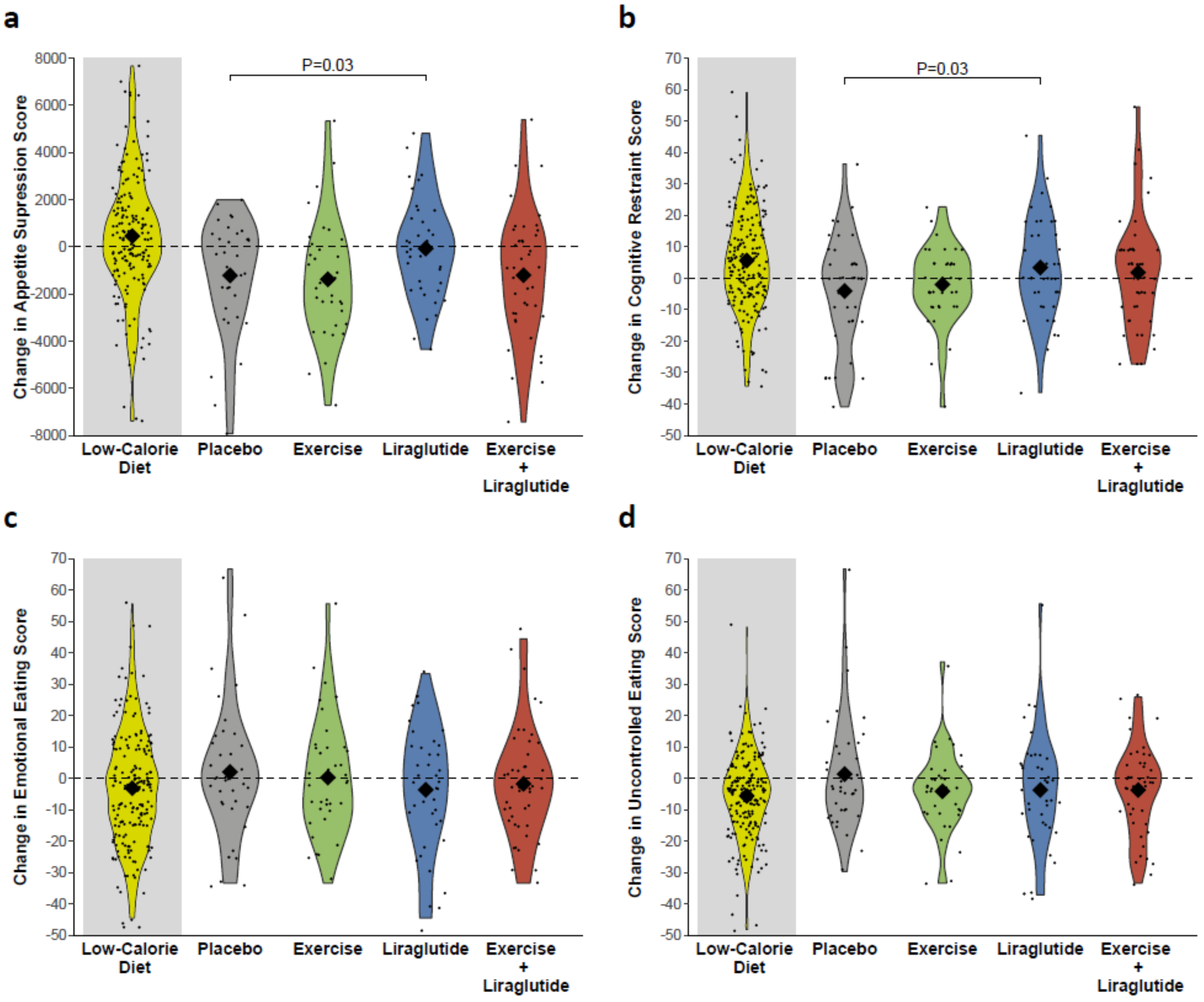

Figure S3. Changes in postprandial appetite suppression and eating behavior during the study in the intention-to-treat population.

Violin plots of observed changes in **a** postprandial appetite suppression score, **b** cognitive restraint, **c** emotional eating, and **d** uncontrolled eating. Changes are from week −8 to week 0 (low-calorie diet, yellow color in shaded area, n=195) for all groups combined and from week 0 to week 52 (after randomization) in the four groups, separately: placebo (grey color, n=40), exercise (green color, n=40), liraglutide (blue color, n=41), and the combination of liraglutide and exercise (red color, n=45). The diamonds indicate observed means, and the dots indicate individual observations. The appetite suppression score was calculated as (satiety + fullness + [100 – hunger] + [100 – prospective food consumption])/4; all were subjective ratings (180 min area under the curve from visual analog scales. Cognitive restraint, emotional eating, and uncontrolled eating were measured on a 0-100 scale by Three-Factor Eating Questionnaire-R18. Results are presented for all randomized study participants (intention to treat population). P-values are provided for significant (P<0.05) differences from the placebo group. P-values are two-sided and unadjusted for multiple analyses and derived from a mixed model analysis. Source data are provided as a Source Data file.

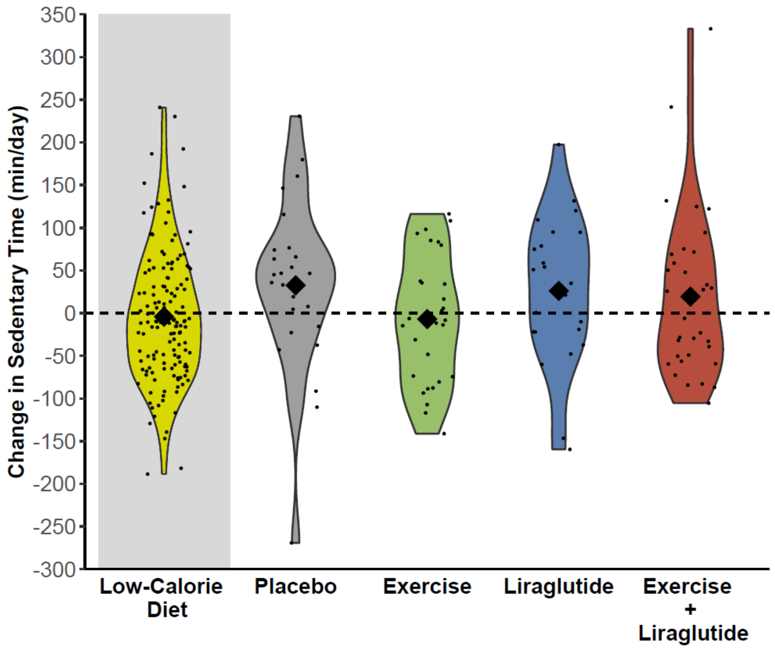

Figure S4. Changes in sedentary time during the study in the intention-to-treat population. Violin plots of observed changes in sedentary time. Changes are from week −8 to week 0 (low-calorie diet, yellow color in shaded area, n=195) for all groups combined and from week 0 to week 52 (after randomization) in the four groups, separately: placebo (grey color, n=40), exercise (green color, n=40), liraglutide (blue color, n=41), and the combination of liraglutide and exercise (red color, n=45). The diamonds indicate observed means, and the dots indicate individual observations. Results are presented for all randomized study participants (intention-to-treat population). Source data are provided as a Source Data file.

**Table S1. Differences in change in outcomes between the three active weight maintenance groups versus placebo from randomization to week 52.**			
	**Exercise vs. placebo**	**Liraglutide vs. placebo**	**Exercise + liraglutide vs. placebo**
	**Estimate (95%CI)**	**Estimate (95%CI)**	**Estimate (95%CI)**
**EATING BEHAVIOR**			
Cognitive restraint (no unit)	3.1 (-5.5; 11.8)	7.2 (-0.7; 15.2)	8.7 (0.3; 17.0)
Emotional eating (no unit)	0.4 (9.6; -10.4)	-5.5 (-14.7; 3.6)	-1.8 (-11.4; 7.9)
Uncontrolled eating (no unit)	-5.1 (-13.5; 3.2)	-5.4 (-13.1; 2.3)	-5.8 (-13.9; 2.4)
**SUBJECTIVE APPETITE RATINGS (FASTING)**			
PFC (mm)	2.1 (-7.2; 11.4)	-1.8 (-10.3; 6.6)	3 (-6.0; 11.9)
Hunger (mm)	5.2 (-7.1; 17.6)	-1.1 (-12.4: 10.2)	2.7 (-9.2; 14.6)
Fullness (mm)	2.7 (-7.6; 13.0)	3.5 (-5.9; 12.9)	4.7 (-5.2; 14.7)
Satiety (mm)	-0.2 (-10.3; 9.9)	10.9 (1.8; 20.1)	1.2 (-8.4; 10.9)
OAS score (mm)	-1.2 (-9.0; 6.6)	4.4 (-2.7: 11.5)	0.1 (-7.4: 7.6)
**SUBJECTIVE APPETITE RATINGS (POSTPRANDIAL)**			
PFC (180 min x mm)	-38 (-1414; 1339)	-1600 (-2842; -359)	-587 (-1896; 722)
Hunger (180 min x mm)	-115 (-1668; 1437)	-1728 (-3130; -327)	-282 (-1761: 1197)
Fullness (180 min x mm)	238 (-1279: 1755)	969 (-399: 2337)	760 (-682: 2203)
Satiety (180 min x mm)	-44 (-1566: 1478)	1195 (-179: 2568)	649 (-800: 2097)
OAS score (180 min x mm)	81 (-1227: 1388)	1374 (194: 2553)	570 (-674: 1814)
**FOOD PREFERENCES**			
High fat sweet, Explicit liking (mm)	3.0 (-6.0: 12.0)	-3.6 (-11.7: 4.5)	3.9 (-4.7: 12.5)
High fat sweet, Implicit wanting (no unit)	0.5 (-7.9: 8.9)	-3.7 (-11.3: 3.9)	-4.4 (-12.5: 3.6)
High fat savory, Explicit liking (mm)	2.1 (-7.4: 11.6)	-4.2 (-12.9: 4.4)	4.7 (-4.4: 13.9)
High fat savory, Implicit wanting (no unit)	-1.5 (-10.7: 7.7)	-4.8 (-13.1: 3.5)	0.8 (-8.0: 9.7)
**ACCELEROMETER-DERIVED PHYSICAL ACTIVITY**			
Sedentary time (min/day)	-23 (-66: 19)	-16 (-58: 26)	-41 (-82: 0)
Light-intensity PA (min/day)	-6 (-31: 19)	-18 (-43: 7)	18 (-6: 42)
MVPA †	1.00 (0.83: 1.2)	0.94 (0.78: 1.13)	1.15 (0.96: 1.38)
**SELF-REPORTED PHYSICAL ACTIVITY**			
Sitting time (min/day)	-38 (-125: 49)	-33 (-109: 44)	-122 (-204: -40)
Walking †	1.00 (0.56: 1.80)	1.35 (0.78: 2.32)	1.07 (0.60: 1.91)
MVPA †	1.27 (0.69: 2.33)	0.71 (0.39: 1.27)	1.10 (0.60: 2.00)

Results are presented for study participants who adhered to the study interventions (per-protocol population) as estimated mean changes (95% CI) from randomization to week 52 as compared with placebo. Values were estimated from a linear mixed model with time, group, sex, age, and a time group interaction as fixed effects.
† Values are estimated geometric mean ratios (below one is a decrease and above one is an increase).
PFC, prospective food consumption; OAS, overall appetite suppression; PA, physical activity; MVPA, moderate-to-vigorous physical activity.

Table S2. Differences in change in outcomes between the three active weight maintenance groups versus placebo from randomization to week 52 in the intention to treat population.			
	**Exercise vs. placebo**	**Liraglutide vs. placebo**	**Exercise + liraglutide vs. placebo**
	**Estimate (95%CI)**	**Estimate (95%CI)**	**Estimate (95%CI)**
**EATING BEHAVIOR**			
Cognitive restraint (no unit)	3.6 (-3.5: 10.8)	7.7 (0.7: 14.7)	5.8 (-1.0: 12.7)
Emotional eating (no unit)	-1.2 (-9.8: 7.4)	-5.2 (-13.6: 3.2)	-3.2 (-11.4: 5.0)
Uncontrolled eating (no unit)	-6.0 (-13.0: 1.1)	-4.4 (-11.3: 2.5)	-5.2 (-11.9: 1.6)
**SUBJECTIVE APPETITE RATINGS (POSTPRANDIAL)**			
PFC (180 min x mm)	99 (-1103: 1300)	-1293 (-2465: -121)	-277 (-1437: 883)
Hunger (180 min x mm)	-125 (-1459: 1208)	-1591 (-2892: -289)	-137 (-1425: 1151)
Fullness (180 min x mm)	123 (-1211: 1456)	902 (-401: 2205)	180 (-1110: 1471)
Satiety (180 min x mm)	-72 (-1402: 1259)	963 (-335: 2262)	125 (-1161: 1410)
OAS score (180 min x mm)	12 (-1129: 1152)	1200 (87: 2313)	168 (-933: 1270)
**FOOD PREFERENCES**			
High fat sweet, Explicit liking (mm)	4.2 (-3.7: 12.1)	-4.0 (-11.6: 3.7)	3.2 (-4.3: 10.8)
High fat sweet, Implicit wanting (no unit)	1.0 (-6.6: 8.7)	-4.8 (-12.2: 2.6)	-4.4 (-11.6: 2.9)
High fat savory, Explicit liking (mm)	1.0 (-7.3: 9.3)	-5.7 (-13.8: 2.4)	6.2 (-1.8: 14.3)
High fat savory, Implicit wanting (no unit)	-1.6 (-9.9: 6.7)	-3.2 (-11.4: 4.9)	3.6 (-4.4: 11.6)
**ACCELEROMETER-DERIVED PHYSICAL ACTIVITY**			
Sedentary time (min/day)	-35 (-77: 6)	-23 (-66: 21)	-13 (-53: 28)
Light-intensity PA (min/day)	-5 (-27: 18)	-12 (-35: 12)	10 (-12: 32)
MVPA †	1.01 (0.85: 1.20)	0.97 (0.81: 1.16)	1.04 (0.88: 1.24)
**SELF-REPORTED PHYSICAL ACTIVITY**			
Sitting time (min/day)	-50 (-131: 31)	-72 (-148: 5)	-87 (-163: -10)
Walking †	1.17 (0.70: 1.93)	1.39 (0.84: 2.30)	1.21 (0.74: 1.99)
MVPA †	1.37 (0.83: 2.27)	0.73 (0.44: 1.22)	1.18 (0.72: 1.94)

Results are presented for all study participants who were randomized (intention-to-treat population) as estimated mean changes (95% CI) from randomization to week 52 compared with placebo. Values were estimated from a linear mixed model with time, group, sex, age, and a time group interaction as fixed effects.
† Values are estimated geometric mean ratios (below one is a decrease and above one is an increase).
PFC, prospective food consumption; OAS, overall appetite suppression; PA, physical activity; MVPA, moderate-to-vigorous physical activity.
