# Supplementary material for: Exploratory analysis of eating- and physical activity-related outcomes from a randomized controlled trial for weight loss maintenance with exercise and liraglutide single or combination treatment
Source: Nat Commun. 2022 Aug 15;13:4770. doi: 10.1038/s41467-022-32307-y (PMC9378667; doi:10.1038/s41467-022-32307-y)
Supplement: Supplementary file 3 — Reporting Summary [file 41467_2022_32307_MOESM3_ESM.pdf]

nature portfolio

Corresponding author(s): Signe Sørensen Torekov

Last updated by author(s): Jun 21, 2022

Reporting Summary

Nature Portfolio wishes to improve the reproducibility of the work that we publish. This form provides structure for consistency and transparency in reporting. For further information on Nature Portfolio policies, see our Editorial Policies and the Editorial Policy Checklist.

Statistics

For all statistical analyses, confirm that the following items are present in the figure legend, table legend, main text, or Methods section.

n/a	Confirmed	
☐	☒	The exact sample size (*n*) for each experimental group/condition, given as a discrete number and unit of measurement
☐	☒	A statement on whether measurements were taken from distinct samples or whether the same sample was measured repeatedly
☐	☒	The statistical test(s) used AND whether they are one- or two-sided<br>*Only common tests should be described solely by name; describe more complex techniques in the Methods section.*
☐	☒	A description of all covariates tested
☐	☒	A description of any assumptions or corrections, such as tests of normality and adjustment for multiple comparisons
☐	☒	A full description of the statistical parameters including central tendency (e.g. means) or other basic estimates (e.g. regression coefficient) AND variation (e.g. standard deviation) or associated estimates of uncertainty (e.g. confidence intervals)
☐	☒	For null hypothesis testing, the test statistic (e.g. *F*, *t*, *r*) with confidence intervals, effect sizes, degrees of freedom and *P* value noted<br>*Give P values as exact values whenever suitable.*
☒	☐	For Bayesian analysis, information on the choice of priors and Markov chain Monte Carlo settings
☒	☐	For hierarchical and complex designs, identification of the appropriate level for tests and full reporting of outcomes
☐	☒	Estimates of effect sizes (e.g. Cohen's *d*, Pearson's *r*), indicating how they were calculated

Our web collection on statistics for biologists contains articles on many of the points above.

Software and code

Policy information about availability of computer code

Data collection	Accelerometer data were collected using the GENEActiv post processing software (v3.2)
Data analysis	SAS Enterprise Guide (v7.15) was used for statistical analyses.<br>The R Package GGIR (v1.11) was used for accelerometer analyses.

For manuscripts utilizing custom algorithms or software that are central to the research but not yet described in published literature, software must be made available to editors and reviewers. We strongly encourage code deposition in a community repository (e.g. GitHub). See the Nature Portfolio guidelines for submitting code & software for further information.

Data

Policy information about availability of data

All manuscripts must include a data availability statement. This statement should provide the following information, where applicable:

- Accession codes, unique identifiers, or web links for publicly available datasets
- A description of any restrictions on data availability
- For clinical datasets or third party data, please ensure that the statement adheres to our policy

Source data are provided with this paper. De-identified data under general data protection regulations (GDPR) may be available for research collaboration purpose upon reasonable request to the corresponding author (Signe Sørensen Torekov, torekov@sund.ku.dk), and will require the completion of a data processing agreement.

Field-specific reporting

Please select the one below that is the best fit for your research. If you are not sure, read the appropriate sections before making your selection.

☒ Life sciences ☐ Behavioural & social sciences ☐ Ecological, evolutionary & environmental sciences

For a reference copy of the document with all sections, see nature.com/documents/nr-reporting-summary-flat.pdf

Life sciences study design

All studies must disclose on these points even when the disclosure is negative.

Sample size	Sample size was calculated based on changes in body weight. For details please see published statistical analysis plan N Engl J Med 2021; 384:1719-1730. DOI: 10.1056/NEJMoa2028198
Data exclusions	No data were excluded from the analyses.
Replication	No replication was performed in this study.
Randomization	Randomization was done in a 1:1:1:1 ratio stratified by sex and age group (<40 years and ≥40 years).
Blinding	The study participants, personnel, and investigators were blinded regarding study medication (liraglutide/placebo treatment) during data collection and analysis of primary endpoint.

Reporting for specific materials, systems and methods

We require information from authors about some types of materials, experimental systems and methods used in many studies. Here, indicate whether each material, system or method listed is relevant to your study. If you are not sure if a list item applies to your research, read the appropriate section before selecting a response.

Materials & experimental systems

n/a	Involved in the study	
☒	☐	Antibodies
☒	☐	Eukaryotic cell lines
☒	☐	Palaeontology and archaeology
☒	☐	Animals and other organisms
☐	☒	Human research participants
☐	☒	Clinical data
☒	☐	Dual use research of concern

Methods

n/a	Involved in the study	
☒	☐	ChIP-seq
☒	☐	Flow cytometry
☒	☐	MRI-based neuroimaging

Human research participants

Policy information about studies involving human research participants

Population characteristics	215 adults with obesity without diabetes were recruited (female, n=135; male, n=80; BMI, 37.0±2.9; age, 42±12 years).
Recruitment	Recruitment was done via newspapers, online media, and flyers from Department of Endocrinology, Hvidovre University Hospital and Department of Biomedical Sciences, University of Copenhagen, Denmark. The intervention duration, exercise program, and comprehensive testing may be potential source of selection bias of individuals who are more likely to adhere to the interventions.
Ethics oversight	The Regional Ethics Committee of the Capital Region of Denmark (H-16027082)

Note that full information on the approval of the study protocol must also be provided in the manuscript.

Clinical data

Policy information about clinical studies

All manuscripts should comply with the ICMJE guidelines for publication of clinical research and a completed CONSORT checklist must be included with all submissions.

Clinical trial registration	EudraCT number, 2015-005585-32; clinicaltrials.gov number, NCT04122716.
Study protocol	The study protocol has been published along with the results on primary outcome here: N Engl J Med 2021; 384:1719-1730. DOI: 10.1056/NEJMoa2028198

Data collection

Study participants were recruited between August 2016 and September 2018. Data was collected until November 2019. Recruitment and data collection was carried out at the Department of Endocrinology, Hvidovre Hospital, Denmark and Department of Biomedical Sciences, University of Copenhagen, Denmark

Outcomes

In this paper, we present results on the following pre-specified secondary outcomes assessed as change from randomization to week 52:
Cognitive restraint, emotional eating, and uncontrolled eating measured by the the three-factor eating questionnaire-R18.
Self-rated prospective food consumption, hunger, fullness, satiety, and overall appetite suppression score measured on a 100 mm visual analog scale in fasted and postprandial state.
Food preferences measured by the Leeds Food Preference Questionnaire.
Physical activity and sedentary time measured by wrist-worn accelerometry (GENEActiv) and the International Physical Activity Questionnaire – Short Form.
Exercise adherence (energy expenditure, duration, intensity) measured with Polar A300 sport watches with heart rate monitors
